# Lipidomic Phenotyping Of Human Small Intestinal Organoids Using Matrix-Assisted Laser Desorption/Ionization Mass Spectrometry Imaging

**DOI:** 10.1021/acs.analchem.3c03543

**Published:** 2023-12-07

**Authors:** Annet A. M. Duivenvoorden, Britt S. R. Claes, Laura van der Vloet, Tim Lubbers, Kristine Glunde, Steven W. M. Olde Damink, Ron M. A. Heeren, Kaatje Lenaerts

**Affiliations:** † Department of Surgery, NUTRIM School of Nutrition and Translational Research in Metabolism, Maastricht University, 6229 ER Maastricht, The Netherlands; ‡ The Maastricht MultiModal Molecular Imaging (M4i) Institute, Division of Imaging Mass Spectrometry (IMS), Maastricht University, 6229 ER Maastricht, The Netherlands; § Department of Surgery, Maastricht University Medical Center+ (MUMC+), 6229 HX Maastricht, The Netherlands; ∥ GROW – School for Oncology and Developmental Biology, Maastricht University Medical Center+ (MUMC+), 6229 HX Maastricht, The Netherlands; ⊥ The Russell H. Morgan Department of Radiology and Radiological Science, Division of Cancer Imaging Research, The Johns Hopkins School of Medicine, Baltimore, Maryland 21205, United States; # The Sidney Kimmel Comprehensive Cancer Center, The Johns Hopkins School of Medicine, Baltimore, Maryland 21205, United States; ∇ Department of Biological Chemistry, The Johns Hopkins School of Medicine, Baltimore, Maryland 21205, United States; ○ Department of General, Gastrointestinal, Hepatobiliary and Transplant Surgery, RWTH Aachen University Hospital, 52074 Aachen, Germany

## Abstract

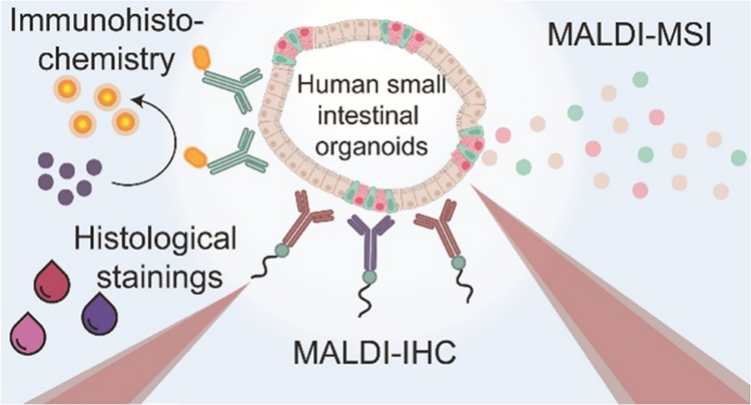

In the past decade, interest in organoids for biomedical research has surged, resulting in a higher demand for advanced imaging techniques. Traditional specimen embedding methods pose challenges, such as analyte delocalization and histological assessment. Here, we present an optimized sample preparation approach utilizing an Epredia M-1 cellulose-based embedding matrix, which preserves the structural integrity of fragile small intestinal organoids (SIOs). Additionally, background interference (delocalization of analytes, nonspecific (histological) staining, matrix ion clusters) was minimized, and we demonstrate the compatibility with matrix-assisted laser desorption/ionization mass spectrometry imaging (MALDI-MSI). With our approach, we can conduct label-free lipid imaging at the single-cell level, thereby yielding insights into the spatial distribution of lipids in both positive and negative ion modes. Moreover, M-1 embedding allows for an improved coregistration with histological and immunohistochemical (IHC) stainings, including MALDI-IHC, facilitating combined untargeted and targeted spatial information. Applying this approach, we successfully phenotyped crypt-like (CL) and villus-like (VL) SIOs, revealing that PE 36:2 [M – H]^−^ (*m*/*z* 742.5) and PI 38:4 [M – H]^−^ (*m*/*z* 885.5) display higher abundance in CL organoids, whereas PI 36:1 [M – H]^−^ (*m*/*z* 863.6) was more prevalent in VL organoids. Our findings demonstrate the utility of M-1 embedding for advancing organoid research and unraveling intricate biological processes within these in vitro models.

## Introduction

Since the establishment of the self-organizing stem cell-driven spheroid and organoid cultures, there has been a surge in their use in biomedical research, overcoming the reliance on traditional cell lines and animal model systems.^1,2^ Organoids are currently one of the most versatile and complex three-dimensional culture systems that replicate tissue heterogeneity and cellular and molecular composition in an in vitro environment.^3−7^ The growing necessity for the comprehensive characterization of diverse organoid models has led to a higher demand for innovative analytical tools. These tools are essential to gain deeper insights into the intricate heterogeneous cellular compositions and molecular processes in organoids. Targeted imaging techniques such as confocal microscopy are widely used for the imaging of organoids. This analytical tool relies on methods involving immunolabeling or fluorescent reporter genes.^8,9^ Confocal microscopy allows for high spatial resolution down to 200 nm^9^ but is limited to only a few preselected targets due to the spectral overlap of the fluorophores.^10^ Therefore, biological information is easily missed in this targeted approach.

Matrix-assisted laser desorption/ionization mass spectrometry imaging (MALDI-MSI) is an analytical tool that detects, localizes, and identifies molecules in an untargeted, label-free manner. Researchers from diverse backgrounds have explored its use in visualizing 3D culture systems, such as spheroids.^11−15^ In contrast, the application of MALDI-MSI for different organoid phenotypes has not yet been thoroughly explored yet. Spheroids are generally cultured in a nonadherent manner (floating), whereas organoid cultures typically rely on a basement membrane extract (BME) for sustaining growth.^7^ Consequently, the isolation of floating spheroids is generally less intricate in comparison to BME-cultured organoids, which reside within the BME matrix and require more intricate isolation methods for ex vivo imaging modalities. A recent study demonstrated a method for the isolation and imaging of lipids in pancreatic ductal cancer organoids (PDAC) with the use of MALDI-MSI.^16^ Although significant effort has been made to demonstrate the utility of MALDI-MSI for imaging lipids in PDACs, its applicability to other, more fragile, organoids models, such as small intestinal organoids (SIOs), has not been demonstrated. Organoid sample preparation for MALDI-MSI requires elaborate washing steps that can compromise the structural integrity of the organoids. Current embedding protocols of organoids could present additional challenges related to analyte delocalization and histological evaluation.^17,18^ As a result, assessment of organoid morphology using hematoxylin and eosin (H&E) staining is hindered due to the stained degraded collagen present in the gelatin-embedding matrix.^19^

An alternative method for embedding and preserving biological specimens is formalin fixation and paraffin embedment (FFPE). Although commonly used for routine clinical diagnostics,^20,21^ the use of FFPE is not ideal for MSI. Besides the possibility of ion suppression from the paraffin, lipids can form a cross-link with formalin, causing class-specific depletion of lipid ion signals in formalin-fixed tissues.^22−24^ Therefore, fresh-frozen material is preferred for MALDI-MSI. Other traditional embedding compounds such as optimal cutting temperature (OCT) matrix are known to interfere with MALDI-MSI experiments.^25^ Recently, a study demonstrated the use of Epredia M-1 embedding matrix (M-1), a cellulose-based embedding medium, to be a suitable substitute for embedding specimens with high water content, such as eyeballs.^26^ Compounds such as M-1 offer exciting prospects for embedding and imaging organoids because of their user-friendly characteristics. Additionally, M-1 embedding could provide a significant advantage over conventional embedding compounds by minimizing the risk of analyte delocalization during sample preparation and ensuring optimal visualization of organoid morphology.

In this study, we aimed to optimize the sample preparation protocol of organoids with M-1 to preserve structural integrity, minimize analyte delocalization, enhance evaluation of organoid morphology, and ensure compatibility with MALDI-MSI. This approach allows the detection and visualization of spatial distributions of lipids in SIOs at a single-cell level (5 μm) in both positive and negative ion polarity. Additionally, M-1 was demonstrated to be compatible with histological and immunohistochemical (IHC) stainings, including MALDI-IHC, where it displayed reduced analyte delocalization and improved (histological) imaging compared to gelatin-embedded samples. This allows for easier coregistration between the images and the possibility of combining untargeted and targeted spatial information. Second, we assessed the applicability of our optimized method for phenotyping crypt-like (CL) and villus-like (VL) human SIOs. This approach allows us to gain valuable insights into cellular heterogeneity and reveal metabolic differences between SIO phenotypes.

## Methods

### Human Intestinal Tissue and Ethics

Healthy intestinal tissue specimens were obtained from a patient (age 69, male) during a pancreaticoduodenectomy at RWTH Aachen University Hospital. The ethics committee of RWTH Aachen approved this study (EK 206/09), and written informed consent was obtained for study participation.

### Organoid Culture and Differentiation

SIOs were generated from isolated intestinal crypts, cultured, and passaged as previously described.^27,28^ Briefly, intestinal crypts were resuspended in basement membrane extract (Geltrex, LDEV-free reduced growth factor basement membrane matrix; Gibco, Carlsbad, CA) and plated in 24-well plates (50 μL of Geltrex per well). Organoids were cultured in growth medium (GM), which consisted of Advanced Dulbecco’s modified Eagle’s medium F12 (Gibco) supplemented with Pen/Strep (50 units/mL penicillin and 50 μg/mL streptomycin) (Gibco), 10 mM HEPES (Gibco), and 1× Glutamax (Gibco), with 1× N2 (Gibco), 1× B27 (Gibco) and 50% (v/v) Wnt3a-conditioned medium, 20% (v/v) Rspondin-1-conditioned medium, 10% (v/v) Noggin-conditioned medium, 10 mM Nicotinamide (Sigma-Aldrich, St. Louis, MO), 50 ng/mL murine EGF (Gibco), 1.25 mM N-acetyl cystein (Sigma-Aldrich), 10 mM Gastrin I (Sigma-Aldrich), 500 nM (TGFβ inhibitor) A83-01 (Sigma-Aldrich), and 10 μM (p38 MAPK inhibitor) SB202190 (Sigma-Aldrich). Differentiation of SIOs was accomplished with differentiation medium (DM), which contained components similar to GM without supplementing the Wnt3a-conditioned medium, Nicotinamide, SB202190, and a 50% reduction of Rspondin-1- and Noggin-conditioned medium. CL organoids were cultured for 12 days in GM medium, whereas VL organoids were cultured for 7 days in GM and 5 days in DM.^28^

### Organoid Isolation, Embedding, and Sectioning

The protocols for collection and embedding SIOs were based on an established protocol for PDAC organoids.^16^ Instructions were followed as described in this protocol for isolating and embedding SIOs. A comparison was made with organoids collected in cell recovery solution (CRS, Corning) instead of 1× phosphate-buffered saline (PBS) (without CaCl_2_ and MgCl_2_, Life Technologies). In short, cell culture medium was aspirated, and 1 mL of cold (4 °C) CRS or 1× PBS was added to each well. Next, the Geltrex domes were gently collected with a plastic Pasteur pipet (Copan Italia 204C), transferred to a 15 mL Eppendorf Tube (Eppendorf), and left incubating on ice (4 °C) for 20 min. Then, all samples were centrifuged for 5 min at 25*g* at 4 °C, and excessive CRS or 1× PBS was aspirated. Excess Geltrex was removed, and organoid pellets were resuspended in 10 mL of 1× PBS, incubated on ice for 20–30 min (inverted every 5 min), and centrifuged for 5 min at 25*g* at 4 °C. These steps were repeated until all Geltrex was removed from the organoid pellet.

The remaining organoid pellet was gently resuspended with 5 mL of cold 50 mM ammonium bicarbonate (ABC, NH_4_CO_3_, Sigma-Aldrich) and centrifuged for 5 min at 25*g* at 4 °C. ABC was aspirated, and organoid pellets were embedded in either preheated (37 °C) 15% gelatin from porcine skin (Sigma-Aldrich) or Epredia M-1 Embedding Matrix (carboxymethylcellulose, M-1, Epredia). Briefly, organoid pellets were resuspended in 400–600 μL of gelatin or M-1 and carefully mixed with a cut off 200 μL pipet tip. The organoid suspensions were transferred to a cryo-mold and covered with a small piece of cork (1.5 mm thick). Gelatin-embedded organoids were frozen in cold isopentane (on dry ice), and the M-1-embedded organoids were snap-frozen in liquid nitrogen (floating, not submerged) for 10 s. Samples were kept on dry ice until they were transferred for storage at −80 °C until further processing.

Additional organoid samples were collected (from each condition) for lipid identification using tandem mass spectrometry (MS^2^). A small volume (50–75 μL) was extracted from the (clean) resuspended organoid pellets in ABC solution and transferred to a 1.5 mL Eppendorf Tube. The samples were centrifuged at 300*g* for 1 min at 4 °C, and ABC solution was aspirated. The remaining pellet was resuspended in 100–150 μL of ABC solution. Organoids were disrupted by manual pipetting and vortexing. A small volume was spotted (50 μL) on a clean indium tin oxide (ITO)-coated glass slide (Delta Technologies Ltd., Loveland, CO) and dried on a heating plate until all liquid was evaporated.

Embedded organoids were sectioned at 12 μm thickness at −14 °C (M-1) and −19 °C (Gelatin) using a cryostat (Microm HM525, Thermo Scientific, Waltham, MA). Organoid sections were thaw mounted onto clean ITO-coated glass slides for MALDI-MSI and adhesive StarFrost glass slides (Knittel Glass, GmbH, Braunschweig, Germany) for (immuno) histological stainings. Tissue slides were stored at −80 °C until further analysis.

### MALDI-MSI and Data Analysis

Before sample preparation, the slides were defrosted in a silica carrier box to avoid condensation. The slides were washed in 50 mM Ammonium formate (4 °C, Sigma-Aldrich) for 15 s in a Petri dish and dried in a desiccator for 30 min. Norharmane matrix (80 mg, Sigma-Aldrich) was sublimed (HTX Sublimator, HTX Technologies, Chapel Hill, NC) at 140 °C for 180 s. MALDI-MSI analysis was performed on a RapifleX MALDI Tissuetyper (Bruker Daltonics GmbH Bremen, Germany) operating in reflectron mode. Data were obtained in positive and negative ion modes with a mass range of *m*/*z* 300–1600 and a pixel size of 5 μm. The laser frequency was set to 5000 Hz, and 100 shots were accumulated at each pixel. Time-of-flight calibration was performed by using red phosphorus. FlexImaging version 5.0 (Bruker Daltonics GmbH, Bremen, Germany) and SCiLS lab 2023c (SCiLS GmbH, Bremen, Germany) were used for processing the imaging data.

### Lipid Identification

MS^2^ experiments were performed on a Q Exactive HF Hybrid Quadrupole-Orbitrap (Thermo Fisher Scientific GmbH, Bremen, Germany) coupled to a MALDI-ESI injector (Spectroglyph, LLC, Kennewick, WA).^29^ Full MS spectra were acquired using 550 ms injection time in both polarities at 1000 Hz and 7.5 Torr (Figures S1 and S2). For each precursor mass, 20 spectra were acquired using 2000 ms injection time while manually moving the MALDI stage at 0.5 mm/s. Precursors were selected using a ±0.5 Da isolation window and fragmented in a higher-energy collisional dissociation cell using a normalized collision energy between 20 and 30 (manufacturer units). All spectra were acquired at a mass resolution of 240,000 (fwhm at *m*/*z* 200), and fragments were matched using the ALEX^123^ lipid calculator. Xcalibur 3.0.16 (Thermo Fisher Scientific GmbH, Bremen, Germany) and mMass v5.5.0 were used for processing the spectra (Figures S3–S7).

### MALDI-IHC

Sections were stained with pan cytokeratin (panCK) PC-MT antibody probe (Ambergen Inc., Boston, MA) according to the manufacturer’s instructions.^30^ Briefly, sections were fixed using 1% paraformaldehyde for 30 min, followed by multiple washing steps with PBS, acetone, and Carnoy’s solution. Sections were rehydrated, followed by incubation with 2% (v/v) normal mouse serum and blocked with a 0.05% octyl β-d-Glucopyranoside, 5% (w/v) bovine serum albumin (BSA) in 1× tris-buffered saline (TBS) for 1 h. Sections were stained with the PC-MT antibody probe with a final concentration of 2 μg/mL overnight at 4 °C in a humidified chamber protected from light. Next, slides were washed with 1× TBS and 50 mM ammonium bicarbonate. The PC-MT was photocleaved by illumination of UV light at 365 nm (Phrozen UV curing lamp, Phrozen Tech Co., Ltd., Hsinchu City, Taiwan) for 10 min (3 mW/cm^2^). 2,5-Dihydroxybenzoic acid (DHB) matrix (50 mg, Sigma) was sublimed (HTX Sublimator, HTX Technologies) at 160 °C for 160 s, followed by recrystallization in an oven (50 °C for 90 s) using a Petri dish containing 0.5% ethanol in water. MALDI-IHC analysis was performed on a RapifleX MALDI Tissuetyper using the reflectron mode in positive polarity. The laser frequency was set to 5000 Hz, and 500 shots were recorded at each pixel. The pixel size was set to 10 μm.

### Immunohistochemistry and Histological Staining

Frozen organoid sections were dried in a desiccator for 10 min, followed by fixation in ice-cold acetone (−20 °C) for 10 min. Tissue slides were rinsed twice in 1× PBS, followed by blocking endogenous peroxidase activity with 0.3% hydrogen peroxide in methanol (H_2_O_2_/MeOH) for 15 min. Next, slides were washed twice in 1× PBS and permeabilized with 0.1% NP-40 (in 1× PBS) for 10 min. The tissue slides were dried and marked with a hydrophobic mini PAP pen (Agar Scientific, Stansted, U.K., Art. No. AGL4197M), and nonspecific antibody binding was blocked with 5% BSA (in 1× PBS) for 30 min. Sections were incubated with the primary antibody overnight at 4 °C. Next, tissue slides were washed twice with 1× PBS and incubated with the biotin-conjugated secondary antibodies for 30 min. This was followed by incubation with an avidin–biotin complex (VectorLabs, Burlingame, CA) for 30 min. Antibody binding was visualized with 3,3′-diaminobenzidine (DAB; Dako, Glostrup, Denmark), and counterstaining was performed with hematoxylin (Merck KGaA, Darmstadt, Germany). Organoid sections were dehydrated and mounted with Entellan (Merck Millipore, Burlington, MA). The antibodies used were Cytokeratin (PanCK, Wide Spectrum, 1:400, Rabbit, Dako) and secondary (biotin-labeled) antibody anti-rabbit (Dako, 1:500).

Alkaline phosphatase staining was used to visualize the brush border enzymes expressed by intestinal enterocytes. Organoid sections were incubated with a mixture of 4-Nitro blue tetrazolium chloride, 4-toluidine salt, and alkaline phosphate buffer and left incubating in a humid chamber at 37 °C for 30 min. Alcian blue staining was used to stain acidic mucins in goblet cells. In short, organoid sections were incubated in 3% acetic acid for 3 min, followed by an Alcian blue solution for 30 min at RT. Both stainings were counterstained with Nuclearfast Red for 5 min and mounted with Entellan. Standard protocols were followed to counterstain for measured MALDI-MSI and MALDI-IHC slides with hematoxylin and Eosin (H&E, Merck KGaA, Darmstadt, Germany). The Aperio CS2 scanner (Leica Microsystems, Amsterdam, The Netherlands) was used for whole slide scanning and digitalization at a 20× magnification.

## Results and Discussion

### Collection and Sample Processing of SIOs

**Figure 1:**
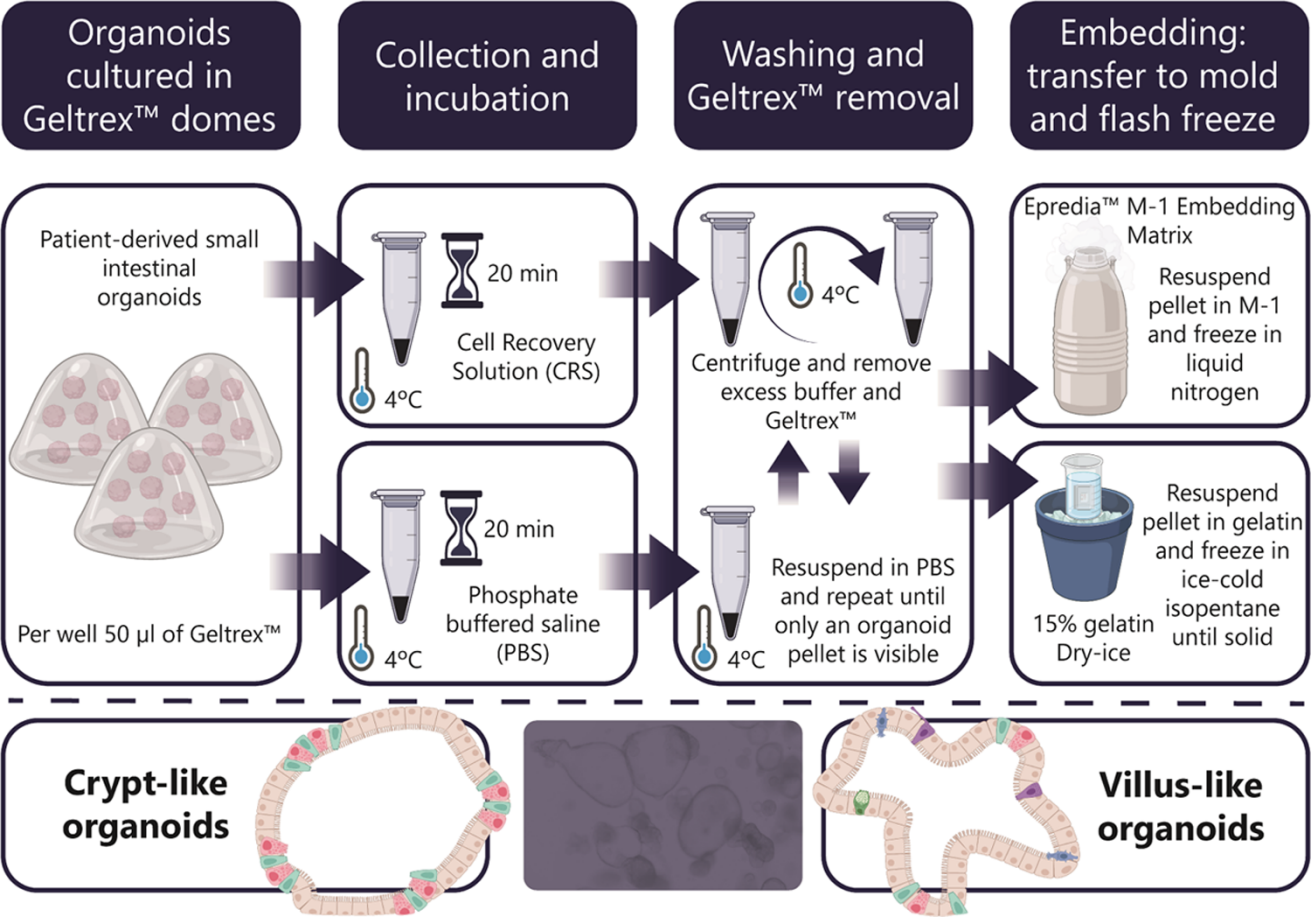
Overview of collection and embedding workflow of human SIOs. The organoids were incubated with either cell recovery solution (CRS) or phosphate-buffered saline (PBS), followed by multiple washing steps to remove the excess buffer and Geltrex. Samples were embedded using either Epredia M-1 embedding matrix or 15% gelatin and snap-frozen.

SIOs are generally cultured in a type of BME containing a unique mix of ECM components and growth factors that allow the cells to grow in a 3D structure (Figure 1). The BME provides the necessary environment for organoid establishment, expansion, and long-term culture but, unfortunately, creates a barrier in sample preparation for MALDI-MSI.^16^ Previously, a novel method was described for isolating and embedding PDAC organoids for lipid identification with MALDI-MSI.^16^ Similar to PDAC organoids, SIOs have a hollow lumen with a thin single-cell layer but contain different cell types. Typical CL SIOs grow as multilobular or cystic organoid structures, mainly containing intestinal stem cells and Paneth cells. For the generation of differentiated or VL SIOs, organoids are cultured in a differentiation medium for 5 days, generating enterocytes and goblet cells.^28^ Therefore, the recovery of SIOs is predicted to differ from that of PDAC organoids based on their size, origin, and morphology.

Our primary aim was to optimize the sample preparation method to improve organoid recovery and preserve their morphology. The protocol presents a significant challenge due to multiple washing and centrifugation steps. Repetitive washing with PBS and centrifugation is necessary to remove excessive BME but could easily damage SIOs due to their fragile structure. An improved approach to prevent this damage includes a short incubation with CRS, which carefully disintegrates the BME (Figure 1). This is followed by a PBS wash and centrifugation at a lower centrifugal force of 25*g* (previously 100*g*). One major advantage of the CRS protocol is its significantly shorter duration compared with the PBS protocol. This not only saves time but also minimizes modification of the original sample.

Next, SIOs were embedded in M-1 and compared to those embedded in a 15% porcine skin gelatin solution. M-1 is a clear and water-soluble solution that allows organoids to be easily embedded and frozen. Furthermore, unlike gelatin solutions, M-1 does not require preheating, which avoids lipid delocalization in the samples or damage of the organoid structure. We compared the original^16^ and adapted protocols to determine their usability for collecting and analyzing SIOs with MALDI-MSI.

### Enhanced Histological Evaluation of SIOs and Improved Lipid Detection via MALDI-MSI

**Figure 2:**
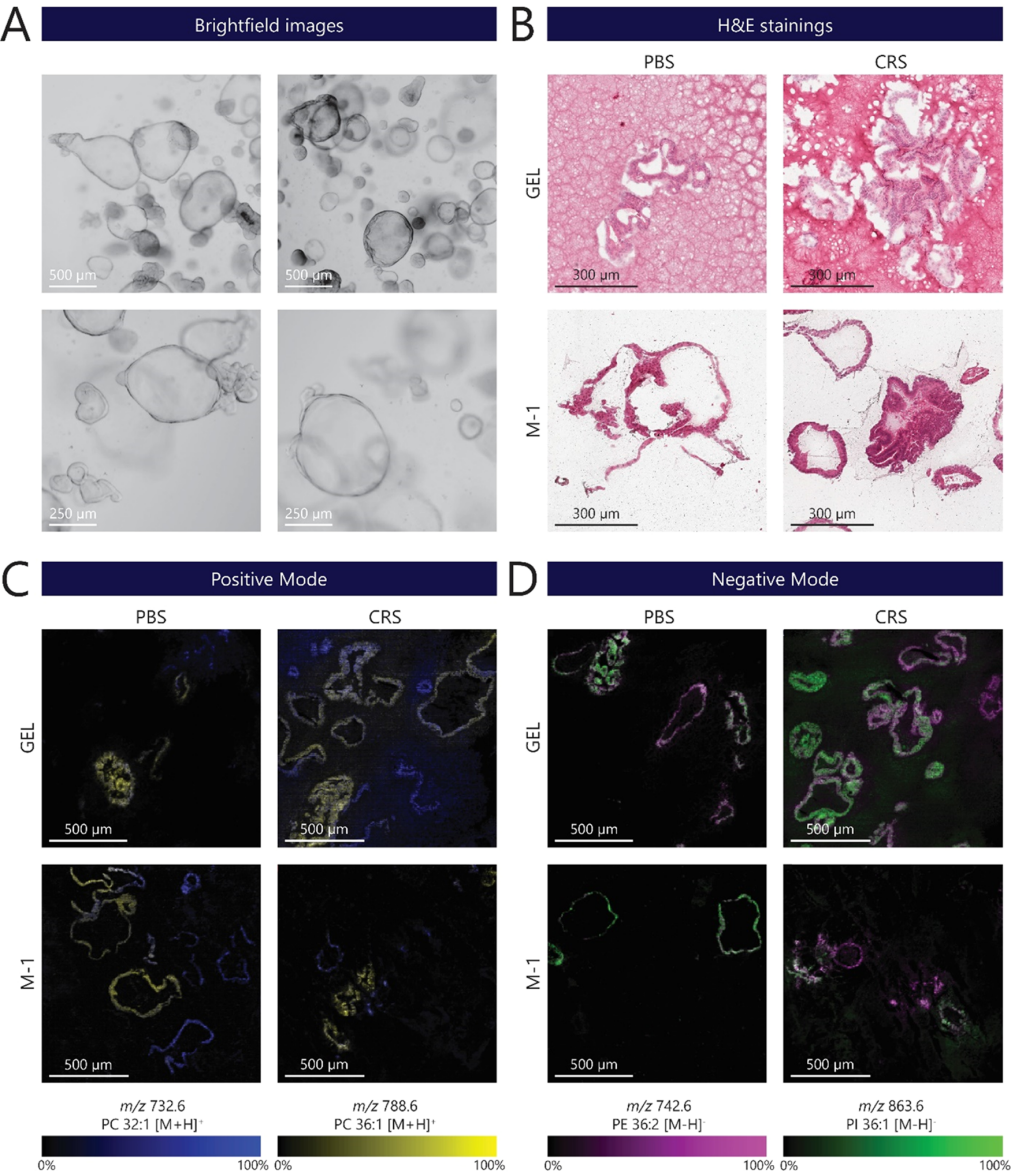
Comparison of small intestinal organoids after PBS or CRS treatment and embedding in gelatin (Gel) or M-1 embedding matrix. (A) Brightfield images representing different phenotypes of 12-day-old human crypt-like (CL) SIOs. The growth of human CL organoids results in a heterogeneous population, with differing sizes among them. Scale bars: 500 μm (top) and 250 μm (bottom) panels. (B) Representative images of embedded organoid sections stained with hematoxylin and eosin (H&E) for different organoid isolation and embedding protocols (scale bar: 300 μm). (C) Heterogeneous distribution of phosphatidylcholines (PC) displayed in CL SIO’s in different sample preparation methods: (blue) *m*/*z* 788.6 (PC 36:1 [M + H]^+^) and (yellow) *m*/*z* 732.6 (PC 32:1 [M + H]^+^) measured in positive ion mode (scale bar: 500 μm). (D) Images obtained in negative polarity revealed heterogeneous distributions of phosphatidylethanolamine (PE) at (pink) *m*/*z* 742.6 (PE 36:2 [M – H]^−^) and phosphatidylinositol (PI) at (green) *m*/*z* 836.6 (PI 36:1 [M – H]^−^). Scale bar: 500 μm. All measurements were conducted on consecutive sections obtained from a single-patient-derived SIO line.

Human SIOs were cultured for 12 days, which resulted in the formation of a heterogeneous population of CL organoids (Figure 2A). A higher yield of organoids and better-preserved organoid morphology in the CRS-treated samples compared to only PBS-treated organoids were obtained (Figure 2B). Histological assessment of gelatin-embedded organoid samples proved to be more challenging due to the simultaneous staining of the gelatin, resulting in a uniformly pink-stained background. This complicates the identification of the single-cell epithelial layer of SIOs in gelatin, which could interfere with future colocalization of morphological organoid structures and lipid profiles. Intriguingly, we found that CRS-treated M-1-embedded samples had a better-preserved organoid morphology, which facilitated an easier distinction of different SIOs features compared to gelatin-embedded samples. As mentioned before, M-1 embedding matrix does not require preheating and is liquid at room temperature. Gelatin-based embedding requires a temperature of 37 °C for liquefaction. During sample collection and removal of excess BME, organoid samples must remain cold (4 °C) to inhibit metabolic activity and prevent degradation. The M-1 embedding matrix proves advantageous because it is readily accessible, affordable, and usable in a straightforward manner, without the need of heating or pretreatment requirements compared to gelatin embedding of SIOs. From the data, it is evident that the utilization of M-1 embedding, as opposed to gelatin, presents a more favorable approach for assessing organoid morphology and streamlining histological coregistration.

MALDI-MSI analysis was performed on adjacent organoid sections to compare lipid detection in both positive and negative polarities (Figure 2C,D). Spectra in positive polarity revealed a heterogeneous distribution of phosphatidylcholines *m*/*z* 732.6 (PC 32:1 [M + H]^+^) and *m*/*z* 788.6 (PC 36:1 [M + H]^+^) in the organoids (Figure 2C). Organoids embedded in gelatin exhibited a higher lipid delocalization compared to that of M-1-embedded organoids (Figure 2C, GEL-CRS). Analyte delocalization is a common problem caused by the sample preparation process.^17,18^ Organoids were washed and centrifuged during sample preparation in an ABC solution. Complete aspiration of the remaining solution is complicated without disrupting the organoid pellet. Hence, the remaining organoid pellet has a high water content due to the remaining BME and ABC solution. This could result in the formation of water droplets and, in combination with heterogeneous heat conduction, cause analyte delocalization.^18,31^ Similar to the positive ion mode, data in negative polarity revealed heterogeneous distributions of phosphatidylethanolamine (PE) at *m*/*z* 742.6 (PE 36:2 [M – H]^−^) and phosphatidylinositol (PI) at *m*/*z* 836.6 (PI 36:1 [M – H]^−^). In addition, distinct analyte delocalization was found in the gelatin-embedded samples, especially in the CRS-treated organoid sample (Figure 2D, GEL-CRS). CRS is a nonenzymatic proprietary solution often used to depolymerize BME matrices. However, the composition of CRS is unknown, and prolonged exposure to organoids might compromise organoid morphology or could cleave membrane and adhesive molecules.^16^ Still, the use of CRS improved the SIO morphology compared to PBS-only washed samples. Nonetheless, it is essential to optimize the CRS incubation for other organoid culture systems to avoid potential structural degradation during sample preparation.

### Characterization of CL and VL Human SIOs by MALDI-MSI

**Figure 3:**
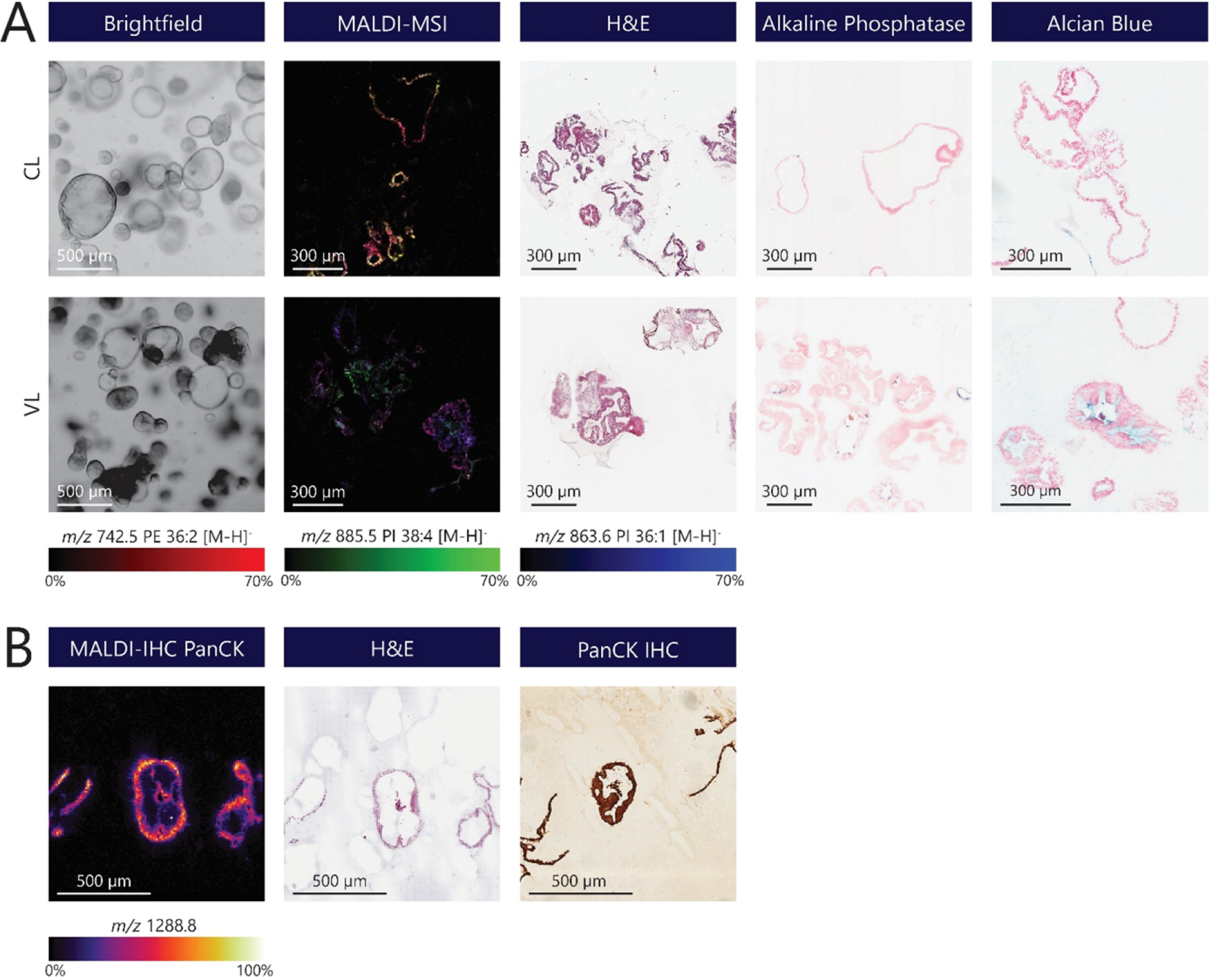
Characterization of human SIOs using MALDI-MSI and (immuno)histochemical staining. (A) Characterization of crypt-like and villus-like human SIOs using MALDI-MSI different histological stainings. Brightfield images representing different phenotypes of human SIOs, including CL and VL organoids (scale bar: 500 μm). MALDI-MSI measurements were performed in negative ion mode, where several lipid species were visualized in both CL and VL SIO: (red) PE 36:2 [M – H]^−^ (*m*/*z* 742.5), (green) PI 38:4 [M – H]^−^ (*m*/*z* 885.5) and (blue) PI 36:1 [M – H]^−^ (*m*/*z* 863.6). Representative stainings for organoid morphology (H&E) and intestinal differentiation markers in CL- and VL-cultured SIO. Alkaline phosphatase staining of the brush border of enterocytes (blue) and Alcian blue-stained goblet cells or mucus (light blue). (B) MALDI-IHC of CL organoids using a PC-MT of PanCK. A consecutive H&E staining was performed on the same sample after measurement. A conservative IHC staining of PanCK (brown) was performed on a consecutive organoid section to validate the MALDI-IHC results (scale bar: 500 μm).

We examined the applicability of our optimized isolation and embedding method (M-1, CRS) to distinguish different SIO phenotypes. CL and VL organoids represent different cellular compositions and functions within the intestinal epithelium, making them a valuable tool for investigating specific aspects of intestinal biology and disease modeling.^28^ In short, CL and VL organoids were generated and isolated after 12 days of culture (Figure 3A).^28^ CL organoids comprised a predominantly cystic population with a visually clear organoid lumen, whereas VL organoids had a more lobular structure with a darker lumen containing cell debris (Figure 3A, brightfield). Additionally, VL organoids had a denser and darker morphology in comparison to CL organoids. We performed MALDI-MSI in negative ion mode, where several lipid species were visualized and identified (Figure 3A, MALDI-MSI). Here, PE 36:2 [M – H]^−^ (*m*/*z* 742.5) and PI 38:4 [M – H]^−^ (*m*/*z* 885.5) showed a more pronounced abundance in CL organoids compared to heterogeneous levels in VL organoids. In addition, PI 36:1 [M – H]^−^ (*m*/*z* 863.6) was more abundant in the VL organoids. These results show that the new isolation and embedding method allows analysis of different lipids classes at high spatial resolution in different SIO phenotypes. Next, a series of histochemical stainings were applied on consecutive slides to confirm the differentiation state of the organoids. H&E staining revealed that most CL organoids consisted of a single-cell epithelial layer. On the other hand, VL organoids exhibited a more distinct morphology resembling villus-like structures, characterized by a thicker epithelial cell layer (Figure 3A, H&E staining). A positive staining of the brush border enzyme alkaline phosphate (purple) confirmed the presence of mature enterocytes in VL organoids, which were absent in CL organoids (Figure 3A, alkaline phosphatase). Alcian blue staining revealed mucus-containing goblet cells and a visible mucus disposition in the lumen of only the VL-organoid (light blue) (Figure 3A, Alcian blue). These findings align with previously published studies^28^ and confirm the successful differentiation from CL to VL and limited diffusion of cellular components. Overall, these data indicate that the adapted method for SIOs is applicable for MALDI-MSI analysis and is suitable for additional histochemical stainings. Moreover, MALDI-MSI enables us to reveal and explore previously undetected metabolic differences between and within various SIO phenotypes in a spatial manner.

MALDI-MSI is generally applied in an untargeted manner. Recent developments within the field of MSI demonstrated a novel technique combining untargeted molecular profiling of MALDI-MSI with targeted IHC (MALDI-IHC).^30,32^ Here, antibodies are labeled with photocleavable mass tags (PC-MTs) that can be detected with MALDI-MSI. MALDI-IHC is especially interesting for various organoid model systems because of the combination of MSI and conventional microscopy on a single organoid section. Here, we investigated the application of this novel technique to SIOs at a single-cell level (10 × 10 μm). CL organoids were stained with a PanCK antibody labeled with a PC-MT (*m*/*z* 1288.8) that can be targeted with MALDI-MSI (Figure 3B). PanCK antibodies are a cocktail of several antibodies that can target a broad spectrum of keratins and detect different epithelial cell types (independent of the origin of the tissue/cell).^33^ PanCK expression was detectable in CL organoids and colocalized with the single epithelial cell lining of the organoid by H&E staining. A conventional IHC staining of PanCK on a consecutive organoid section validated our findings of PanCK expression by MALDI-IHC. These findings demonstrate that multimodel MALDI-IHC can be applied for organoid-based studies and complements untargeted MALDI-MSI. Additionally, the sample preparation protocol developed in this study does not interfere with this method. However, further research is required before multiplex MALDI-IHC can be performed with multiple cell-specific targets. The current protocol includes tissue fixation with formalin, which requires antigen retrieval by heat-induced epitope retrieval (HIER). However, HIER does not always result in an optimal epitope recovery and risks a loss of organoid sections. Therefore, an additional coating of ITO slides with poly-l-lysine is suggested to improve the adhesion of organoid sections, or further optimization of the fresh-frozen MALDI-IHC protocol could potentially allow the formalin fixation step to be omitted.

## Conclusions

In this study, we introduce an enhanced protocol for the isolation and embedding of human SIOs. The organoid yield was increased, and the sample preparation time was shortened with the use of the CRS protocol. M-1 embedding matrix was demonstrated to be a suitable embedding material compatible with both MSI and immuno(histological) stainings, where it showed reduced analyte delocalization, optimized histological visualization of organoid morphology, and absence of nonspecific antibody binding compared to existing gelatin-based embedding protocols. This protocol showed compatibility with untargeted lipid MALDI-MSI analysis, as well as other complementary analytical techniques such as IHC, histochemical staining, and MALDI-IHC. Furthermore, the protocol allows the detection of a broad range of glycerophospholipid species in both positive and negative ion modes and reveals different lipid distributions and heterogeneity among human SIOs. Differences in lipid expression were visualized among CL and VL organoids, revealing metabolic differences between the organoid phenotypes. This highlights the strength of using MSI for studying lipid metabolism and phenotyping of organoid models, including the increased capability to combine MSI with histological staining for spatial correlation.

## Supporting Information Available

Spectra of crypt-like versus villus-like organoids in positive ion mode (Figure S1); spectra of crypt-like versus villus-like organoids in negative ion mode (Figure S2); MS/MS spectrum of PC 32:1 (Figure S3); MS/MS spectrum of PC 36:1 (Figure S4); MS/MS spectrum of PE 36:1 (Figure S5); MS/MS spectrum of PI 36:1 (Figure S6); and MS/MS spectrum of PI 38:4 (Figure S7) (PDF)
