# Supplementary material for: Lipidomic Phenotyping Of Human Small Intestinal Organoids Using Matrix-Assisted Laser Desorption/Ionization Mass Spectrometry Imaging
Source: Anal Chem. 2023 Dec 7;95(50):18443–50. doi: 10.1021/acs.analchem.3c03543 (PMC10733903; doi:10.1021/acs.analchem.3c03543)
Supplement: Supplementary file 1 — ac3c03543_si_001.pdf [file ac3c03543_si_001.pdf]

Supporting information:

Lipidomic phenotyping of human small intestinal organoids using matrix-assisted laser desorption/ionization mass spectrometry imaging

Annet A.M. Duivenvoorden[1#], Britt S.R. Claes[2#], Laura van der Vloet[2], Tim Lubbers[3,4], Kristine Glunde[5,6,7], Steven Olde Damink[1,3,8], Ron M.A. Heeren[2*], and Kaatje Lenaerts[1*]

1. Department of Surgery, NUTRIM School of Nutrition and Translational Research in Metabolism, Maastricht University, 6229 ER Maastricht, The Netherlands
2. The Maastricht MultiModal Molecular Imaging (M4i) Institute, Division of Imaging Mass Spectrometry (IMS), Maastricht University, 6229 ER Maastricht, The Netherlands
3. Department of Surgery, Maastricht University Medical Center+ (MUMC+), 6229 HX Maastricht, The Netherlands
4. GROW – School for Oncology and Developmental Biology, Maastricht University Medical Center+ (MUMC+), 6229 HX Maastricht, The Netherlands
5. The Russell H. Morgan Department of Radiology and Radiological Science, Division of Cancer Imaging Research, The Johns Hopkins School of Medicine, Baltimore, MD 21205, United States
6. The Sidney Kimmel Comprehensive Cancer Center, The Johns Hopkins School of Medicine, Baltimore, MD 21205, United States
7. Department of Biological Chemistry, The Johns Hopkins School of Medicine, Baltimore, MD 21205, United States
8. Department of General, Gastrointestinal, Hepatobiliary and Transplant Surgery, RWTH Aachen University Hospital, 52074 Aachen, Germany

[#] These authors have equally contributed

* Corresponding authors:
Prof. Ron M.A. Heeren
Email: r.heeren@maastrichtuniversity.nl
Dr. Kaatje Lenaerts
Email: k.lenaerts@maastrichtuniversity.nl

Table of Contents

Figure S1: Crypt-like *versus* villus-like organoid spectra in positive ion mode S2
Figure S2: Crypt-like *versus* villus-like organoid spectra in negative ion mode...........S2
Figure S3: MS/MS spectrum of *m/z* 732.6 identified as PC 32:1 $[M+H]^+$...................S3
Figure S4: MS/MS spectrum of *m/z* 788.6 identified as PC 36:1 $[M+H]^+$...................S3
Figure S5: MS/MS spectrum of *m/z* 742.5 identified as PE 36:1 $[M-H]^-$......................S4
Figure S6: MS/MS spectrum of *m/z* 863.6 identified as PI 36:1 $[M-H]^-$ S4
Figure S7: MS/MS spectrum of *m/z* 885.6 identified as PI 38:4 $[M-H]^-$ S5

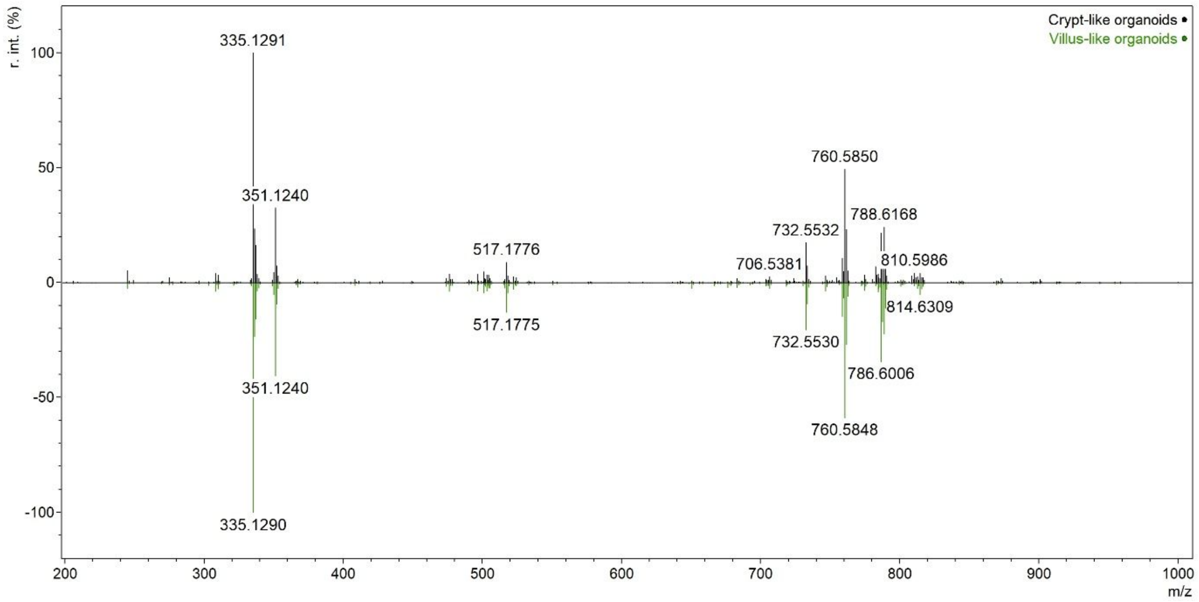

Figure S1: Comparison of crypt-like organoid (top, black) *versus* villus-like organoid spectra (bottom, green) in positive ion mode.

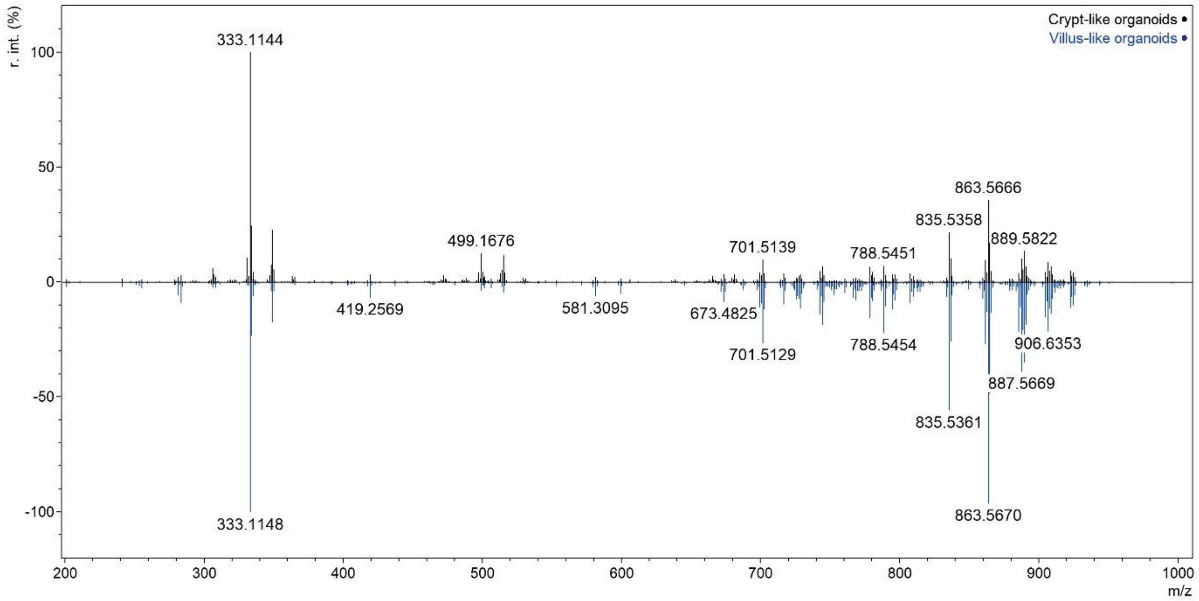

Figure S2: Comparison of crypt-like organoid (top, black) *versus* villus-like organoid spectra (bottom, blue) in negative ion mode.

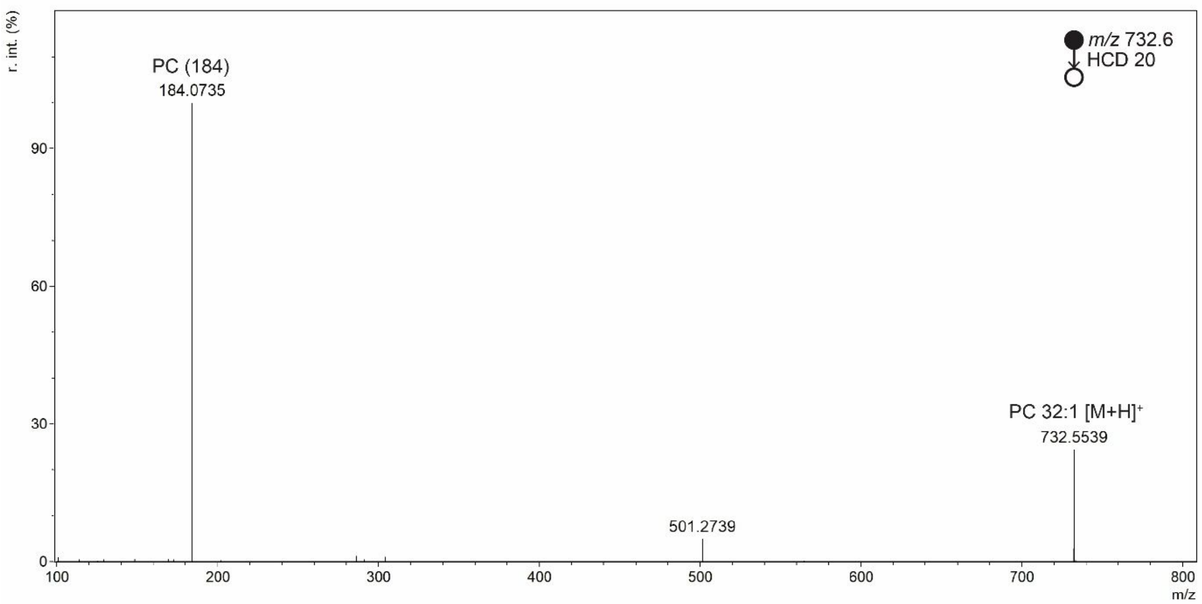

Figure S3: MS/MS spectrum of *m/z* 732.6 identified as PC 32:1 $[M+H]^+$.

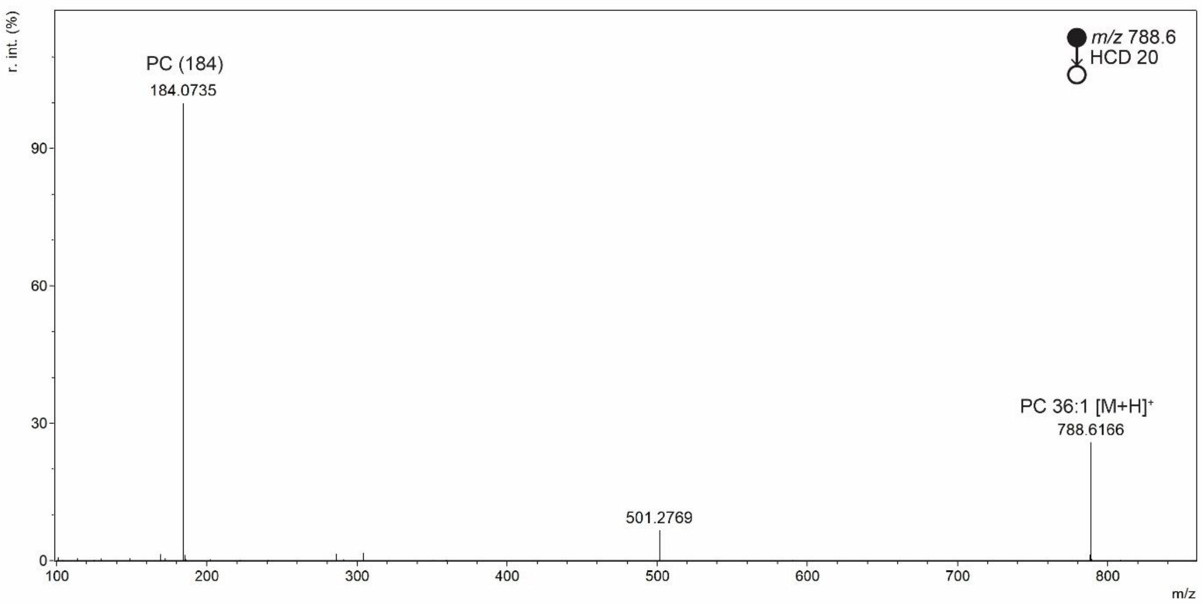

Figure S4: MS/MS spectrum of *m/z* 788.6 identified as PC 36:1 $[M+H]^+$.

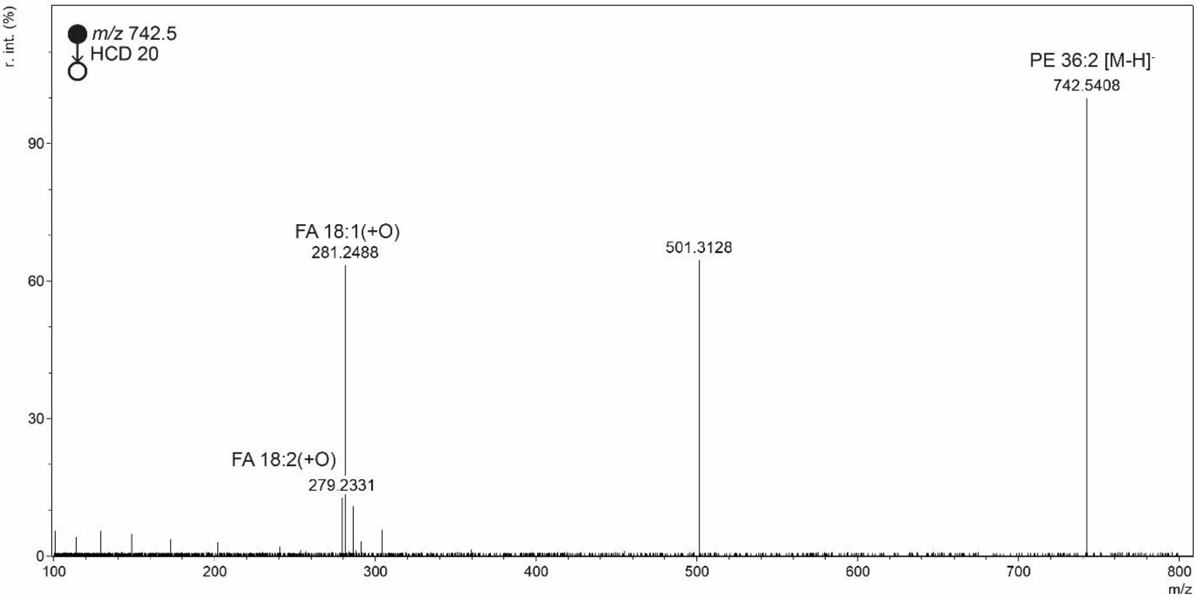

Figure S5: MS/MS spectrum of m/z 742.5 identified as PE 36:1 [M-H]-.

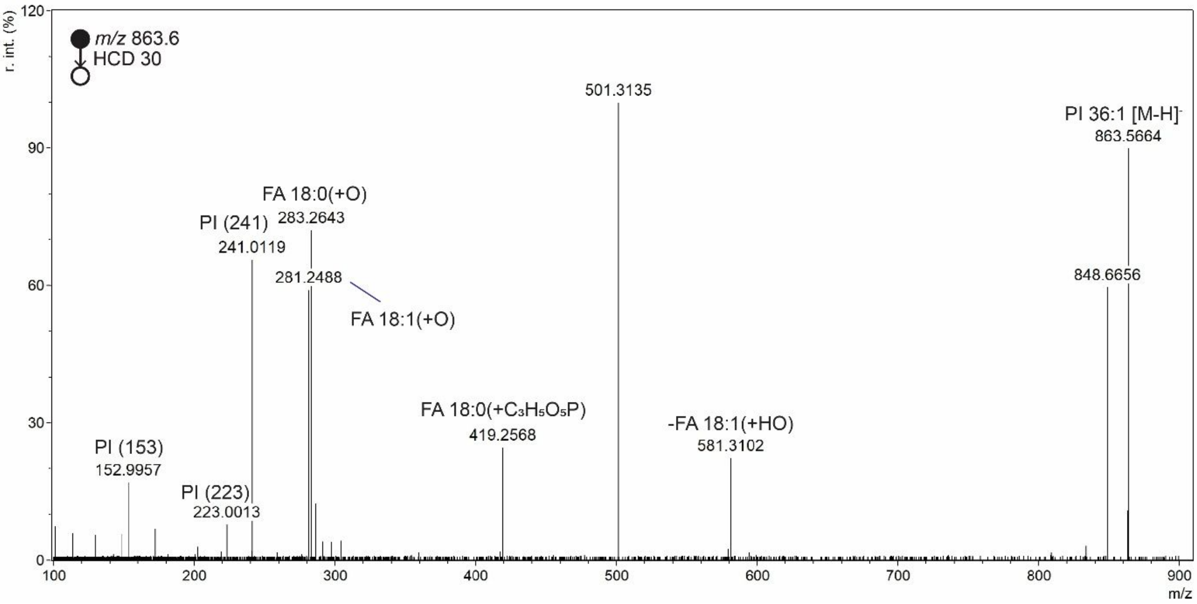

Figure S6: MS/MS spectrum of *m/z* 863.6 identified as PI 36:1 [M-H]-.

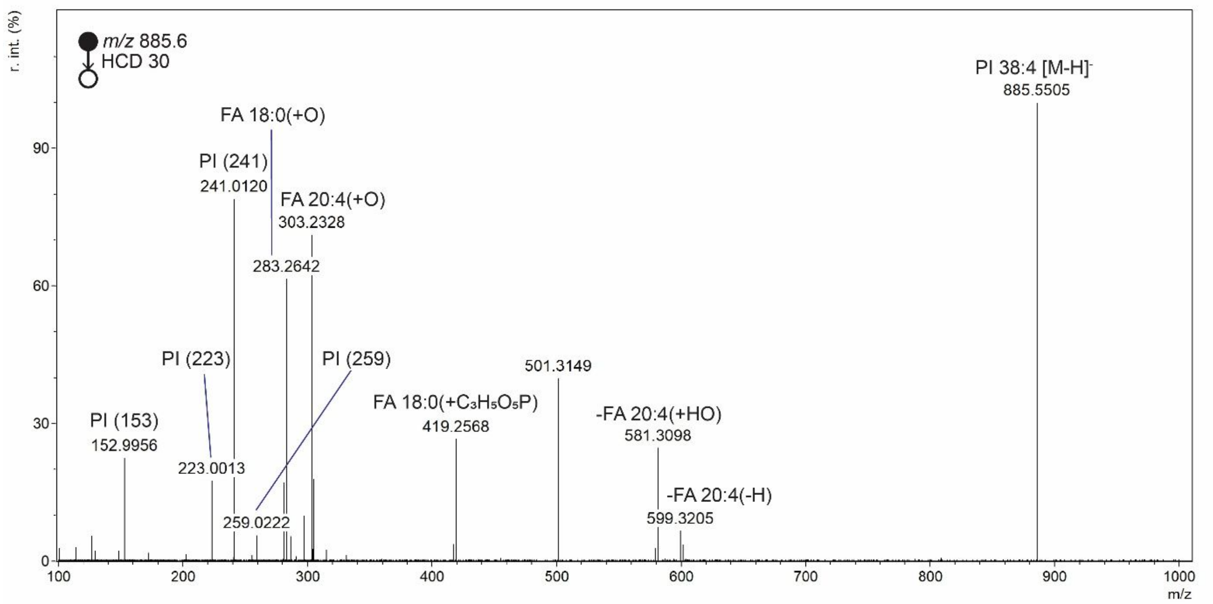

Figure S7: MS/MS spectrum of *m/z* 885.6 identified as PI 38:4 [M-H]$^-$.
